# Supplementary material for: Supported MOCVD TiO2 Thin Films Grown on Modified Stainless Steel Mesh for Sensing Applications
Source: Nanomaterials (Basel). 2023 Sep 29;13(19):2678. doi: 10.3390/nano13192678 (PMC10574371; doi:10.3390/nano13192678)
Supplement: Supplementary file 1 [file nanomaterials-13-02678-s001.zip › nanomaterials-2599382-supplementary.pdf]

Supplementary Material

Table S1. Composition of the acid etching solutions (all the used acids were freshly opened).

Acid solutions 10M (50 mL)	
HCl	42 mL (conc. HCl) + 8 mL (H2O)
H_2SO_4	28 mL (conc. H2SO4) + 22 mL (H2O)
HCl + H_2SO_4	21 mL (conc. HCl) + 14 mL (conc. H2SO4) + 15 mL (H2O)

Table S2. Physical-chemical properties, costs of the single acids (based on Sigma-Aldrich quotations), and the respective solution costs (for 50 mL solution at a 10M).

Physical-chemical properties and costs of the employed acids				
Acid	Density (g/cm3)	Molecular Weight (g/mol)	Molarity (mol/L)	Cost[1] (€/L)
Conc. HCl	1.19	36.5	12	75.6
Conc. H_2SO_4	1.83	98	18	49.6
**Cost of 50 mL solution at 10M**				
Acid solution			Cost[1] (€/50 mL)	
HCl + H_2O			3.18	
H_2SO_4 + H_2O			1.39	
HCl+ H_2SO_4 + H_2O			2.28	

[1]Exclude the costs of deionized water for the solution. The cost of the reagents is referred to in September 2023 Sigma-Aldrich quotations.

Table S3. Element atomic percentage from XPS analyses in relation to the different acid etching solutions.

	**C 1s at.%**	**O 1s at.%**	**Cr 2p at.%**	**Fe 2p at.%**
SS	56.9	36.6	1.4	5.1
SS-AT-HCl	66.3	28.2	2.7	2.9
SS-AT-H_2SO_4	59.3	32.9	3.9	3.9
SS-AT-HCl/H_2SO_4	73.8	22.3	1.8	2.1

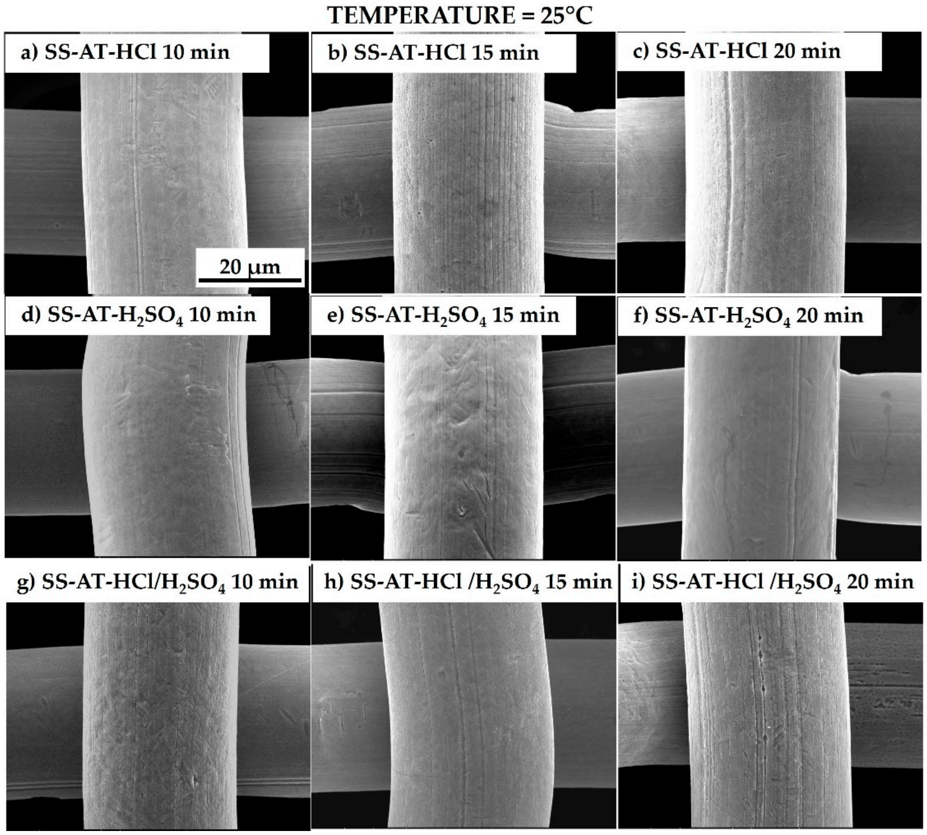

Figure S1. SEM micrographs of the meshes etched at 25°C with the HCl solution 10M for 10, 15, and 20 min (a, b, c), with the H_2SO_4 solution 10M for 10, 15, and 20 min (d, e, f), and with the HCl/H_2SO_4 solution 10M for 10, 15, and 20 min (g, h, i).

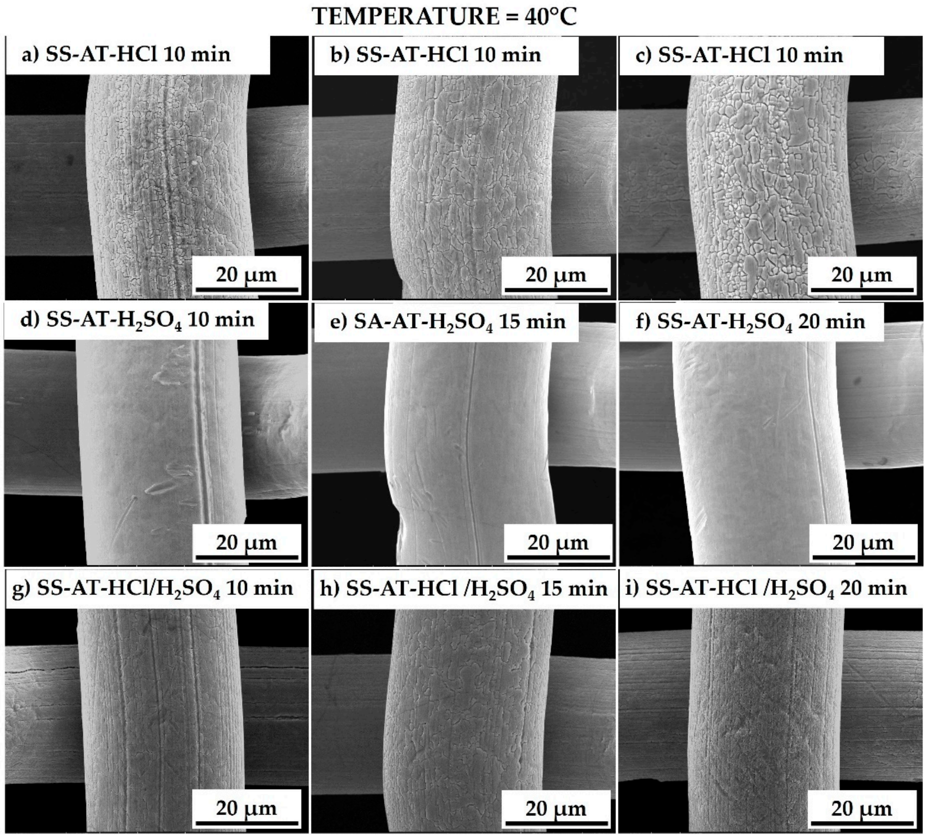

Figure S2. SEM micrographs of the meshes etched at 40°C with the HCl solution 10M for 10, 15, and 20 min (a, b, c), with the H_2SO_4 solution 10M for 10, 15, and 20 min (d, e, f,), and with the HCl/H_2SO_4 solution 10M for 10, 15, and 20 min (g, h, i).

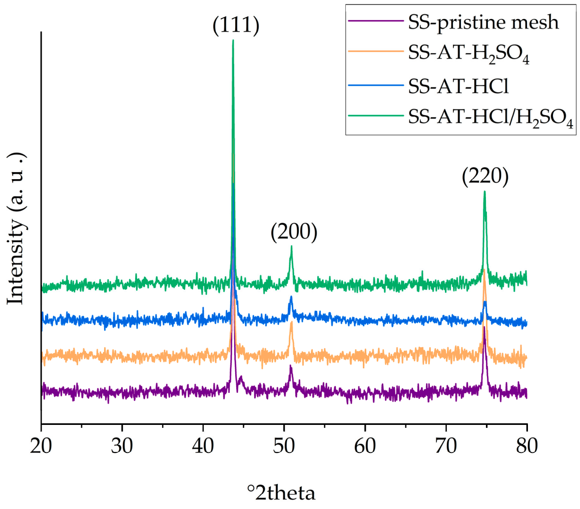

Figure S3. X-ray diffraction patterns of AISI 316 stainless-steel meshes before and after the etching treatments with HCL, H_2SO_4, and HCl/ H_2SO_4 acid solutions (10M) at 55°C for 15 min.

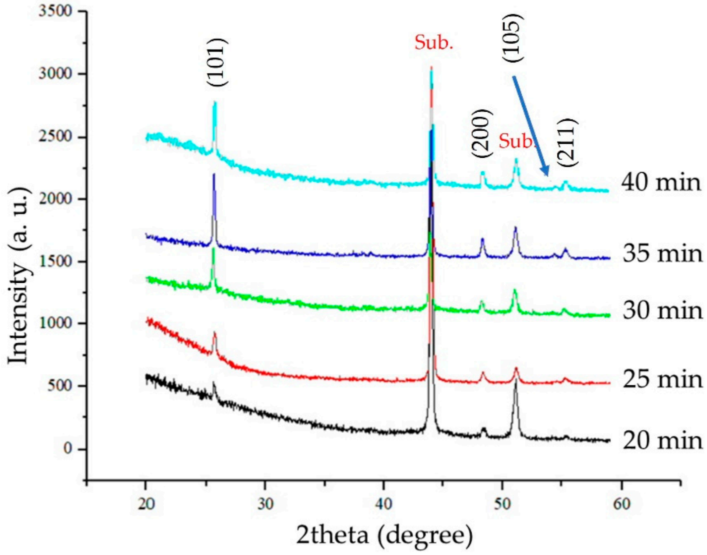

Figure S4. XRD patterns of TiO_2 thin films deposited via MOCVD on acidified networks (SS-AT-TiO_2) after different growth times: 20, 25, 30, 35, and 40 mins (with sub = acidified-mesh substrate).

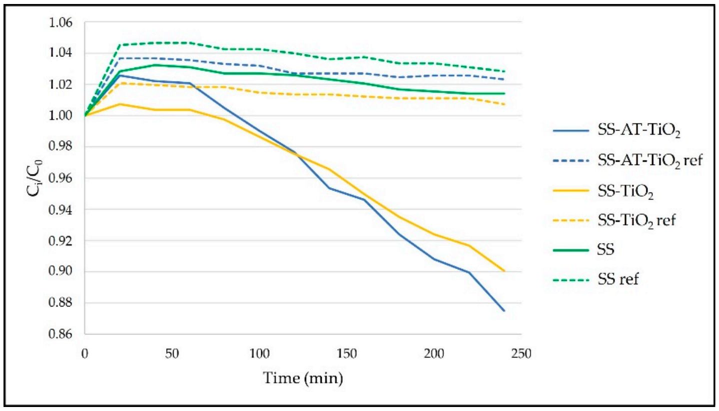

Figure S5. Normalized trend (at $[MB]_{ti}/[MB]_{t0}$) versus time for the stainless-steel mesh (green), stainless-steel mesh with TiO_2 deposition (yellow), and acid-treated mesh with 33 min of TiO_2 deposition (blue). Solid lines = samples, dotted lines = references (i.e., data obtained by keeping the solutions in the dark). UVA lamp was turned on at t = 60 minutes.
